# The reduced genomes of Parcubacteria (OD1) contain signatures of a symbiotic lifestyle

**DOI:** 10.3389/fmicb.2015.00713

**Published:** 2015-07-21

**Authors:** William C. Nelson, James C. Stegen

**Affiliations:** Microbiology, Biological Sciences Division, Pacific Northwest National LaboratoryRichland, WA, USA

**Keywords:** Parcubacteria, genomics, symbiosis, pan-genome, genome reconstruction, candidate phyla, groundwater, streamlining

## Abstract

Candidate phylum OD1 bacteria (also referred to as Parcubacteria) have been identified in a broad range of anoxic environments through community survey analysis. Although none of these species have been isolated in the laboratory, several genome sequences have been reconstructed from metagenomic sequence data and single-cell sequencing. The organisms have small (generally <1 Mb) genomes with severely reduced metabolic capabilities. We have reconstructed 8 partial to near-complete OD1 genomes from oxic groundwater samples, and compared them against existing genomic data. The conserved core gene set comprises 202 genes, or ~28% of the genomic complement. “Housekeeping” genes and genes for biosynthesis of peptidoglycan and Type IV pilus production are conserved. Gene sets for biosynthesis of cofactors, amino acids, nucleotides, and fatty acids are absent entirely or greatly reduced. The only aspects of energy metabolism conserved are the non-oxidative branch of the pentose-phosphate shunt and central glycolysis. These organisms also lack some activities conserved in almost all other known bacterial genomes, including signal recognition particle, pseudouridine synthase A, and FAD synthase. Pan-genome analysis indicates a broad genotypic diversity and perhaps a highly fluid gene complement, indicating historical adaptation to a wide range of growth environments and a high degree of specialization. The genomes were examined for signatures suggesting either a free-living, streamlined lifestyle, or a symbiotic lifestyle. The lack of biosynthetic capabilities and DNA repair, along with the presence of potential attachment and adhesion proteins suggest that the Parcubacteria are ectosymbionts or parasites of other organisms. The wide diversity of genes that potentially mediate cell-cell contact suggests a broad range of partner/prey organisms across the phylum.

## Introduction

The Parcubacteria, also known as Candidate Phylum OD1 bacteria (OD1), were originally identified by phylogenetic analysis of 16S rRNA genes amplified from a variety of environmental samples (Harris et al., 2004). The environments from which these bacteria were observed were exclusively anoxic. The first hint at the biology of the Parcubacteria came from a single sequenced BAC clone from Zodletone Spring, which revealed a few metabolic genes supporting an anaerobic lifestyle (Elshahed et al., 2005). More recently, extensive metagenomic sequencing of DNA from anoxic groundwater samples at the Rifle, CO Integrated Field Research Challenge (IFRC) site has yielded several near-complete genome sequences of diverse Parcubacteria and a single full-length genome sequence (Wrighton et al., 2012; Kantor et al., 2013). In addition, a single-cell genomics effort focused specifically on uncultured phyla provided nine additional partial Parcubacterial genomes from samples taken at widely varying, yet all anoxic, environments (Rinke et al., 2013).

Metabolic reconstruction efforts on the known Parcubacteria genome sequences (Wrighton et al., 2012, 2014; Kantor et al., 2013) indicated sparse mechanisms for energy and nutrient conservation. Members are non-respiring, lacking genes for the tricaboxylic acid cycle (TCA) and electron transport, leading to the proposal that Parcubacteria obligately ferment simple sugars to organic acids, although some are apparently capable of degrading complex carbon sources such as cellulose and chitin. They have also been implicated in hydrogen and sulfur cycles in anoxic sediments (Wrighton et al., 2012, 2014). The Parcubacterial genomes generally lack genes for biosynthesis of amino acids, nucleotides, vitamins, and lipids. In spite of this, they also have a limited number of transport systems, calling into question how they acquire these essential metabolites (Kantor et al., 2013).

**Figure 1:**
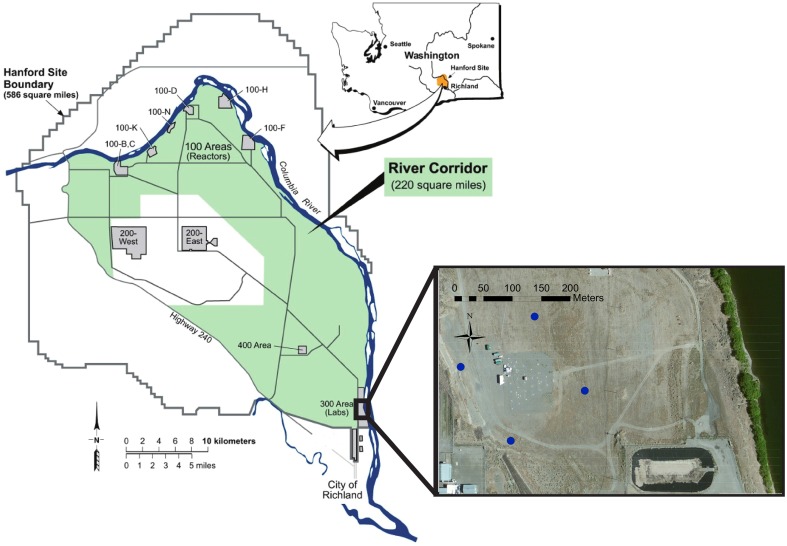
**Study site map**. Map showing the location of the Hanford site within the state of Washington and the location of the four corner wells (blue markers) surrounding the IFRC within the 300 Area.

The Hanford 300 Area is an unconfined aquifer containing an extensive and persistent uranium plume resulting from disposal of nuclear fuel fabrication wastes from 1943 to 1994 (Zachara et al., 2013). Similar to Rifle, CO, the 300A is the location of an IFRC site that has been investigating reactive mass transfer and biogeochemical processes controlling U(VI) concentrations in a linked vadose zone-groundwater-river system. The site is adjacent to the Columbia River (Figure 1) which experiences large variations in river stage associated with seasonal mountain snowpack dynamics. As a result of the large river stage variations the groundwater in the 300A is subject to changes in elevation level and even flow reversals that can alter chemical composition, flow velocity, and microbial community dynamics (Lin et al., 2012b). Previous investigations of community dynamics revealed the presence of Parcubacteria in the oxic portion of the Hanford 300 A aquifer (Lin et al., 2012a,b) that prompted this more detailed investigation into properties of these members of candidate phylum OD1.

Metagenomic sequencing and genome reconstruction has yielded eight near-complete Parcubacterial genomes from microbial communities present in Hanford 300A groundwater. Genome comparison against publicly available Parcubacterial genome sequences has determined a core set of genes for the phylum, and identified genes specific to the organisms found in the oxic and anoxic environments. In addition, the Parcubacteria apparently lack several genes that are highly conserved across other known bacterial species. Examination of the Parcubacterial genome sequences for proposed signatures for free-living, streamlined organisms and symbiotic, parasitic, and commensal organisms found similarities and differences with both groups. We propose that OD1 organisms are ectosymbionts or parasites attached to the external surfaces of other microbial cells to facilitate access to nutrients and energy sources produced by the host.

## Materials and methods

### Well-sampling, DNA prep, and sequencing

Samples were taken in December 2011 from Hanford 300 Area IFRC well 399-1-60 (46.3711 N 119.2759 W). Between 80 and 120 L of groundwater were pumped through a 47 mm diameter 1.2 μm pre-filter before being filtered through a 142 mm diameter, polyethersulfone 0.2 μm filter (Pall Corporation, part number 60305); without pre-filters the 0.2 μm filter clogged rapidly; pre-filters were replaced as necessary to maintain flow. The pre-filters were discarded and the 0.2 μm filters were transported to the laboratory on wet ice within sterile containers and stored at −80°C prior to DNA extraction.

DNA was extracted from filters using a modification of methods presented in Bostrom et al. (2004). Filters were removed from −80°C, further frozen with liquid-N, crushed, and transferred to sterile 15 mL tube. Filter material was incubated at 85°C for 10 min in 8 ml of lysis buffer, as defined in Bostrom et al. (2004). The solution was cooled slowly to avoid DNA fragmentation, and lysozyme was added to achieve a final concentration of 1 mg mL^−1^; the solution was incubated at 37°C for 30 min. SDS was then added to a final concentration of 1% and proteinase-K was added to a final concentration of 100 μg mL^−1^; the solution was incubated at 55°C for approximately 12 h. The sample was then centrifuged for 5 min at 1500 × g to pellet the filter paper, and the supernatant was decanted into a 50 ml sterile tube, followed by isopropanol precipitation and pellet elution in 50 μL TE. The sample was then treated with 5 μL of 10 mg mL^−1^ RNase at 37°C for 30 min, followed by isopropanol precipitation and elution in 100 μL TE. Resulting DNA was shipped on dry ice to Los Alamos National Laboratory for shotgun sequencing; for each sample, one Illumina TruSeq DNA library was generated and was then sequenced on two lanes of the Illumina HiSeq 2000 platform using the 2 × 100 paired-end chemistry. Metagenomic sequence is available from Genbank under SRA entry SRX1041926.

### Genome reconstruction

Raw read files were evaluated for quality using FastQC v0.10.1(available from http://www.bioinformatics.babraham.ac.uk/projects/fastqc/). Reads were trimmed using Trimmomatic (Bolger et al., 2014), using ILLUMINACLIP 2:30:10, MINLEN 40 and SLIDINGWINDOW 10:15. Of 28,405,919 input read pairs, 23,985,145 remained after trimming. Assembly was performed using IDBA_ud v1.1.0 (Peng et al., 2012) with default parameters resulting in 253,569 scaffolds with a N50 of 1245 and a total length of 237 MB. Scaffolds > 5 kb in length (3580 totaling 50 Mb) were considered further. Coverage values were determined by searching the assembled read set against the scaffolds set using bowtie2 (Langmead and Salzberg, 2012) and using the samtools depth function (Li et al., 2009) to calculate per-base depth and a custom script to calculate average coverage across the scaffold. AMPHORA2 (Wu and Scott, 2012) was used to identify and estimate taxonomy for phylogenetic marker genes. Tetranucleotide frequencies were calculated for each scaffold using a script provided by the Banfield laboratory (I. Sharon, personal communication), using a window size of 5000 nt and this data was used to construct an emergent self-organizing map (ESOM), as previously described (Dick et al., 2009). The ESOM map was evaluated using both the coverage values and estimated phylogeny, and scaffolds were segregated into initial bins.

Binned sequences were evaluated for consistency of coverage, %G+C content and estimated taxonomy, and for specificity by enumerating a set of 105 conserved single-copy genes (CSCG), as described in Rinke et al. (2013). Bins containing multiple copies of any CSCG were examined to determine if scaffolds were misplaced. CSCG results were also used to estimate completeness of genomic complement.

Reconstructed genome sequences have been deposited at DDBJ/EMBL/GenBank under the following accessions: LFCK00000000 (C7867-001), LFCL00000000 (C7867-002), LFCM00000000 (C7867-003), LFCN00000000 (C7867-004), LFCO00000000 (C7867-005), LFCP00000000 (C7867-006), LFCQ0000000 (C7867-007), and LFCR00000000 (C7867-008).

### Genome annotation and ortholog analysis

Final genome bins were evaluated for membership in candidate phylum Parcubacteria by analysis of phylogenetic marker genes. Coding genes were identified using prodigal (Hyatt et al., 2010); tRNA genes were identified using tRNAscan-SE (Lowe and Eddy, 1997) and ncRNAs were identified using the HMMER3.0 package (Eddy, 2011) and the Rfam database (Daub et al., 2015). Gene function was predicted using a combination of TIGRfam (Haft et al., 2013) and Pfam (Finn et al., 2014) HMMs, BLAST (Camacho et al., 2009) results against the public Parcubacterial genome data, and results from the RAST genome annotation system (Aziz et al., 2008; Overbeek et al., 2014).

The basis of cross-genome comparison was an ortholog table. Bi-directional best hits were calculated from BLASTp results for all genome pairs. Blast alignment regions had to cover 70% of protein length, and the percent identity had to be within two standard deviations of the average amino acid identity. The global protein set was also clustered using MCL (Enright et al., 2002). Multiparanoid (Alexeyenko et al., 2006) was used to calculate putative ortholog groups. Resulting groups with more than one member per genome were examined to see if gene neighborhood analysis could split the group.

### Core and unique analysis

Pan-genome analysis was performed in a manner similar to Tettelin et al. (2005). Briefly, the predicted protein set from one genome was searched against another using BLASTP. The number of shared proteins and the total number of unique proteins was calculated, using cutoffs of 50% similarity across > 50% of the protein length. The other genomes were added to the analysis one at a time in a random order. To normalize for varying genome size, for each individual analysis, the observed count of shared or total unique proteins was divided by the median protein count of the genomes being analyzed. Analysis was repeated either until exhaustion of possible sequential combinations or 100 trials, and average scores were reported.

### Phylogenetic analysis

Protein sequences were aligned using the linsi module of the MAFFT package (Katoh and Standley, 2014). Alignment columns with >30% gap characters were removed. For concatenated protein trees, alignments were concatenated at this step. RAxML v 7.3.0 (Liu et al., 2011) was used to generate maximum likelihood trees, using algorithm “a,” the PROTGAMMAJTT model, and 100 alternative runs. Trees were visualized using the Archaeopteryx module of the forester package (https://code.google.com/p/forester/).

## Results and discussion

### Reconstruction of OD1 genomes from a metagenomic sample

**Figure 2:**
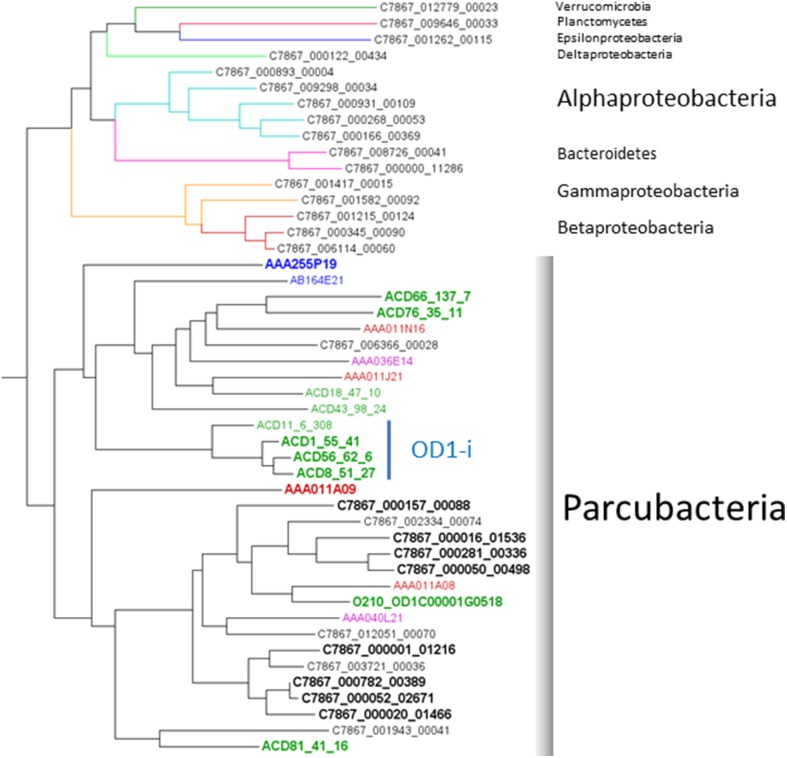
**Phylogeny of RplB sequences discovered on C7867 scaffolds**. A single deeply-branching Thaumaracheal sequence is not displayed. Edge colors denote estimated taxonomy, indicated by phylum names to the right. Node label colors indicate the source of the sampled DNA, black; Hanford IFRC, green; Rifle IFRC, blue; Lake Sakinaw, red; Homestake Mine drainage. Boldface indicates sequences from reconstructed genomes used in the comparison.

OD1 genomes were reconstructed from a metagenomic sample derived from groundwater communities sampled from a well adjacent to the IFRC (C7867) screened from 6-18 m. Approximately 35% of the paired-end reads assembled into 253,569 scaffolds with an N50 of 1245. Scaffolds longer than 2 kb (*N* = 17,044) were screened for phylogenetic marker genes using AMPHORA2 (Wu and Scott, 2012). The markers were used to derive an estimated taxonomy for the scaffolds containing marker genes. Phylogenetic analysis of RplB sequences identified shows that 40% of all RplB sequences identified within the assembly set are from OD1 bacteria (Figure 2). Previous community surveys performed within the IFRC have shown that Parcubacteria are usually in low abundance, with the summed relative abundance of all Parcubacterial OTUs being under 2% of the total population, however, sporadic blooms have been observed bringing relative abundance of individual OTUs to >14% (Lin et al., 2012b). The current result likely reflects both the serendipitous capture of a Parcubacterial bloom at the sample site and bias in the assembly process favorable to assembly of OD1 genomic sequence, perhaps due to its phylogenetic distance from other organisms present in the data set. Scaffolds containing marker genes assigned to OD1 were used to probe emergent self-organizing maps based on tetranucleotide content to generate putative genome-specific bins. Bins were both checked for specificity and scored for completeness of genomic information by assessing their complement of conserved single-copy genes (CSCG) (as in Rinke et al., 2013). Previous work on Parcubacteria has reported that they have very small genomes that must either lack certain genes conserved in most other bacteria, or contain instead divergent orthologs that are not easily recognizable (Kantor et al., 2013). As such, the gene complement of the complete OD1 RAAC4 genome was used as the standard for completeness. From the C7867 metagenome, 8 partial OD1 genomes (C7867-001 to C7867-008) were obtained with estimated completeness ranging from 69% to 97% (Table 1). The genomes have average read coverage ranging from 8- to 42-fold. Despite this moderate coverage, the genomes assembled well, ranging from 3 to 29 scaffolds in each bin. Genome sizes are small, with estimates for complete genome length ranging from ~600 to 900 kb, and G+C content ranging from 36% to 56%.

**Table 1:** **Genomic data used in comparison**.

**Source**	**Genome**	**Scafs**	**Total bp**	**%GC**	**Compl (%)**	**RelCompl (%)^a^**
Hanford	C7867-001	5	812,017	53	73	89
Hanford	C7867-002	29	631,749	44	78	96
Hanford	C7867-003	29	529,551	38	56	68
Hanford	C7867-004	6	778,726	56	79	97
Hanford	C7867-005	3	563,550	42	76	93
Hanford	C7867-006	8	652,563	36	70	86
Hanford	C7867-007	8	702,407	48	74	90
Hanford	C7867-008	6	808,014	48	74	91
Homestake Mine	AAA011-A09	28	388,659	37	59	72
Lake Sakinaw	AAA255-P19	29	553,464	39	61	75
Rifle	ACD1	69	1,295,178	38	81	100
Rifle	ACD56	73	912,614	40	64	79
Rifle	ACD66	143	621,261	41	56	68
Rifle	ACD76	84	900,453	43	81	99
Rifle	ACD8	53	991,308	38	78	96
Rifle	ACD81	98	959,320	43	72	89
Rifle	RAAC4	1	693,530	31	81	100

a *Using the RAAC4 gene complement as the comparison standard*.

### Comparative genome analysis

The reconstructed genomes were compared against the complete RAAC4 genome and eight other partial reconstructed OD1 genomes–six from the Rifle site (Wrighton et al., 2012; Kantor et al., 2013), and two from the “Microbial Dark Matter” project's Homestake Mine drainage and Sakinaw Lake samples (Rinke et al., 2013) (Table 1). Genomes were selected for phylogenetic diversity (see Figure 2), and had to be greater than 65% complete (by the completeness criteria described above) and contain on average only one copy of each CSCG (an indication of monospecific binning). Ortholog groups were constructed across the 17 genomes, clustering the 13,594 genes into 1905 families with two or more members (comprising 9542 genes), and 4052 genes with no apparent ortholog.

### The conserved core of candidate phylum parcubacteria

**Figure 3:**
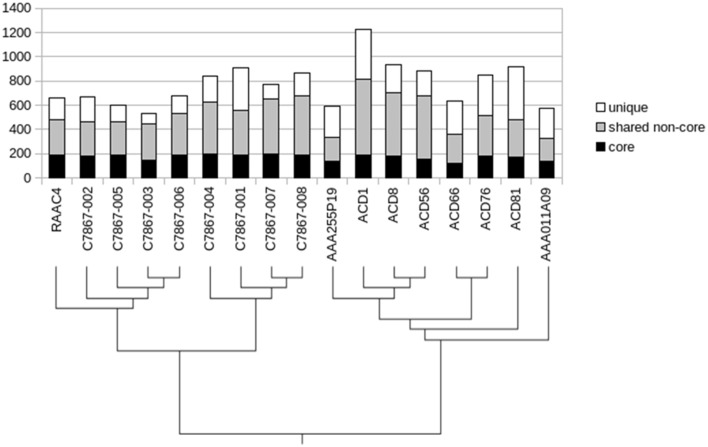
**Distribution of gene conservation across the Parcubacteria genomes**. Shaded subsections represent degree of conservation across all genomes, with the lowest black section representing genes conserved in 13–17 genomes, the gray section conserved in 2–12 genomes, etc. to the top white section representing genes unique to a single organism.

Determination of conserved genes across various taxonomic ranks can provide information about the evolutionary history of a lineage (Zhang and Sievert, 2014), thus we examined the ortholog families to define a core genome for the OD1 phylum. Since most of the genome sequences are incomplete, genes present in 13 or more of the 17 genomes were considered part of the core (Figure 3). A set of 202 ortholog families was identified, comprising 18–31% of the estimated total gene complement of OD1 genomes. Over half of the core genes also had a conserved position adjacent to at least one other core gene. Many of the rest appear to have a conserved position within their respective sub-groups [i.e., the OD1-i group or the C7867 clade (see Figure 2)]. Core genes included DNA replication functions, cell division proteins, transcription machinery, translation machinery, protein folding and trafficking genes, and genes for peptidoglycan biosynthesis (Table S1). Few genes for biosynthesis of amino acids or nucleotides were part of the core, and only nine genes in the core have no known function, limiting the number of functions that may be present as novel genes. Although most genes central to glycolysis are conserved, enolase and pyruvate kinase were only identified in 10 and 11 of the genomes, respectively. A conserved gene cluster contains activities central to the non-oxidative branch of the pentose phosphate pathway. The genes for a type IV pilus and competence proteins ComEC, ComF, and DprA (also known as Smf) are also in the conserved core.

### Shared non-core and unique genes

Genes not in the core reflect the evolutionary history of the sublineages and adaptations by each organism to its particular environment and lifestyle (Tettelin et al., 2005; Grote et al., 2012; Zhang and Sievert, 2014). These genes can be separated into those that are present in multiple organisms (shared non-core) and those that are unique to a specific organism (Grote et al., 2012). Between the shared non-core genes and the unique genes, there are 9436 genes in the “flexible” genome of the Parcubacteria. COG categories overrepresented in the flexible genome (relative to the core genome) include energy production and conversion, amino acid transport and metabolism, nucleotide transport and metabolism, lipid transport and metabolism, cell wall/membrane/envelope biogenesis, secondary metabolites, signal transduction, and defense mechanisms (Table 2). These functions are typical of those known to vary between species (Zhang and Sievert, 2014), and also be carried by various mobile genetic elements (Rankin et al., 2011).

**Table 2:** **COG category breakdown for core, shared non-core and unique gene sets**.

**COG category**	**Core**	**SNC**	**Unique**
A RNA processing and modification	–	–	1
B Chromatin structure and dynamics	–	1	–
C Energy production and conversion	–	43	43
D Cell cycle control, cell division, chromosome partitioning	9	7	27
E Amino acid metabolism and transport	2	20	88
F Nucleotide metabolism and transport	4	30	68
G Carbohydrate metabolism and transport	8	25	54
H Coenzyme metabolism and transport	1	23	32
I Lipid metabolism and transport	–	9	24
J Translation, ribosomal structure, and biogenesis	87	32	57
K Transcription	11	26	126
L Replication, recombination, and repair	24	54	164
M Cell wall/membrane/envelope biogenesis	14	59	311
N Cell motility	–	1	1
O Post-translational modification, protein turnover, chaperones	10	43	74
P Inorganic ion transport and metabolism	2	15	47
Q Secondary metabolite biosynthesis, transport, and catabolism	–	3	12
R General function prediction	13	88	227
S Function unknown	11	105	190
T Signal transduction	2	24	74
U Intracellular trafficking and secretion	7	7	13
V Defense	1	16	48
Multiple assignments	19	67	258

### Aerobic metabolism

**Figure 4:**
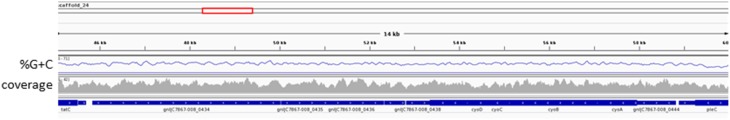
**Coverage and %G+C of C7867-008 scaffold region containing CytO genes**. %G+C was calculated across 120 nt windows with a step size of 30 nt. IGV (Thorvaldsdottir et al., 2013) was used to display the data.

Examining the genomes of putatively free-living organisms endemic to an oxic environment, we expected to find evidence of aerobic metabolism, which had not previously been identified in an OD1 genome. Intriguingly, only 3 of the 8 C7867 genomes contained genes suggesting the capability of using O_2_ as a terminal electron acceptor. Genomes C7867-007 and C7867-008 each contain all four subunits of cytochrome bo(3) ubiquinol oxidase (CytO). We believe the attribution of CytO to these bins to be correct because the genes: (1) had coverage and composition values consistent with other regions of the scaffolds (Figure 4) and other scaffolds in the bins; (2) are located at equivalent locations in the middle of long contigs in each of the independently-assembled bins; and (3) phylogenetic trees of the concatenated protein sequences of the CytO subunits (Figure S1) and a valyl-tRNA ligase (Figure S2) found on the same scaffold show similar branching patterns. In *Escherichia coli*, CytO is the primary respiratory oxidase under high oxygen tension. A membrane-bound, quinone-dependent NAD(P)H dehydrogenase passes electrons to ubiquinone, which then shuttles them to CytO (Dinamarca et al., 2002). CytO then reduces O_2_ to H_2_O and pumps protons across the cytoplasmic membrane to generate proton motive force (PMF), which can then be used to generate ATP through the F_1_F_0_ ATP synthase. Although F_1_F_0_ ATP synthase is not part of the OD1 core genome, being present in only 10 of the 17 genomes examined, the C7867-007 and C7867-008 genomes both contain complete gene clusters for the F_1_F_0_ ATP synthase. No membrane-bound, quinone-dependent NAD(P)H dehydrogenases were identified in either genome, nor are there any apparent quinone biosynthesis genes.

The C7867-001 genome has cytochrome bd-I ubiquinol oxidase (CytD) subunits A and B [the small CydX subunit (VanOrsdel et al., 2013) could not be identified, and is likely absent]. These genes, like the CytO genes mentioned above, also assembled centrally on a long scaffold, and had consistent read coverage and composition with the rest of the scaffold sequence (data not shown). The two subunits are known to have different evolutionary rates, so phylogenetic evaluation was performed both separately and as a concatenated alignment. Their positions within the resulting trees do not match that of the RpoB gene found elsewhere on the scaffold. This is not unexpected, however, since lateral gene transfer is thought to have played a role in the evolutionary history of this gene (for a review, see Borisov et al., 2011). CytD has a distinct structure and cofactor requirement from the more common heme-copper oxidases (HCO). All known members oxidize quinols to reduce O_2_, build PMF via transmembrane charge separation rather than proton pumping. CytD has been implicated in a variety of physiological processes including O_2_ scavenging, aromatic compound degradation, resistance to nitrosative, alkaline, hydrostatic and temperature stresses, and providing the oxidizing power for disulfide bond formation. Similar to C7867-007 and C7867-008, C7867-001 possesses a complete F_1_F_0_ ATP synthase gene cluster and lacks any quinone biosynthesis genes.

Other genes were identified that were absent from the C7867 genomes but present in most of the other OD1 genomes. These include: a protein involved in formation of a pentaglycine bridge in peptidoglycan, a second copy of DNA repair gene *uvrA*, thioredoxin *trxA* (although *trxB* is in the core), an archaeal-type phosphoglucosamine mutase (involved in UDP-GlcNAc biosynthesis), and polyribonucleotide nucleotidyltransferase (PNP), a 3′-5′ exoribonuclease (Li and Deutscher, 1994). A BLAST search of these proteins against the unbinned contigs did identify proteins with 55–70% amino acid sequence identity, suggesting an origin of a closely-related species, however the contigs on which they resided had lower coverage values (5-8X) and thus may derive from other lower-abundance parcubacterial community members. PNP is a component of the RNA degradosome, a multisubunit complex involved in both tRNA processing and mRNA degradation (Gorna et al., 2012). Its composition varies across species, but always includes RNase E, an endoribonuclease, RNA helicase RhlB, and PNP. These other common components of the RNA degradosome are not identifiable in any of the genomes in the comparison. The degradosome has been implicated in fast metabolic response to changes in growth conditions (Carpousis, 2007), thus its absence could reflect a lifestyle of slow, steady growth.

### Novel strategies for genome reduction

The genes in the set of conserved single-copy genes (CSCG) used for completeness analysis (see Methods) are assumed to be part of a universal functional core and essential for bacterial viability (Rinke et al., 2013). Some of the genes considered CSCGs are entirely absent from the 17 OD1 genomes in this analysis (Table S2). For those genes, a more detailed analysis was undertaken to assess whether it is reasonable to conclude that these genes are actually absent from these genomes, and not just in the assembly gaps. Specifically, we searched for mechanisms that the Parcubacteria might have evolved to compensate for the lack of these genes.

**Figure 5:**
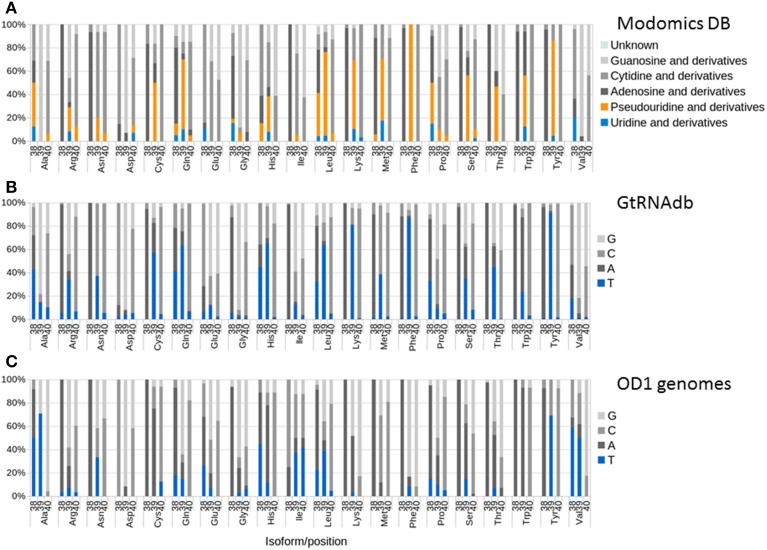
**Nucleotide usage and modification at positions 38, 39, and 40 of tRNAs**. For each isotype, the three columns represent positions 38, 39, and 40 (in relation to *E. coli* standard). Color sections represent nucleotide and modification data. **(A)** Modomics nucleotide and modification data; **(B)** GtRNAdb nucleotide data; **(C)** OD1 nucleotide data.

Pseudouridine synthase A (PSA), encoded by the *truA* gene, modifies uridine bases at positions 38, 39, and 40 within the anti-codon stem-loop in tRNAs to enhance stability. The Modomics database (Machnicka et al., 2013) contains sequences of RNAs, including experimentally determined positions and species of modified bases. Analysis of bacterial sequences deposited in the Modomics database reveals variance in the activity of PSA modification at the three positions and between tRNA isotypes. Where modification is observed, it is usually predominant at only one of the three positions, and a majority of the U residues is modified (Figure 5A). Because of the limited dataset available in Modomics (24 species represented, many only partially), we also examined nucleotide usage at tRNA positions 38, 39, and 40 in 630 sequenced genomes in the GtRNAdb (Chan and Lowe, 2009). These results generally agreed with the Modomics analysis, with T found predominantly at positions in isoforms where pseudouridine modification was observed in the Modomics data (Figure 5B), usually position 38 or 39. Examination of predicted tRNAs from OD1 genomes shows that the OD1 populations have reduced T usage in positions 38 and 39 of isoforms that are targets of PSA activity (e.g., tRNA-Lys39, tRNA-Met39, tRNA-Phe39), the exception being tRNA-Tyr39 (Figure 5C). T is preferentially replaced by a C or G residue in most isoforms, which could help stabilize the stem loop structure due to the additional hydrogen bond present in G-C base pairs relative to A-T base pairs. Curiously, T usage is elevated at positions in isoforms that are not targets of PSA, for example tRNA-Ala39, tRNA-Val38, and tRNA-Val39. These results are consistent with the hypothesis that the Parcubacteria have evolved to preclude the necessity for PSA function through altered sequence content in the anti-codon stem loop.

**Figure 6:**
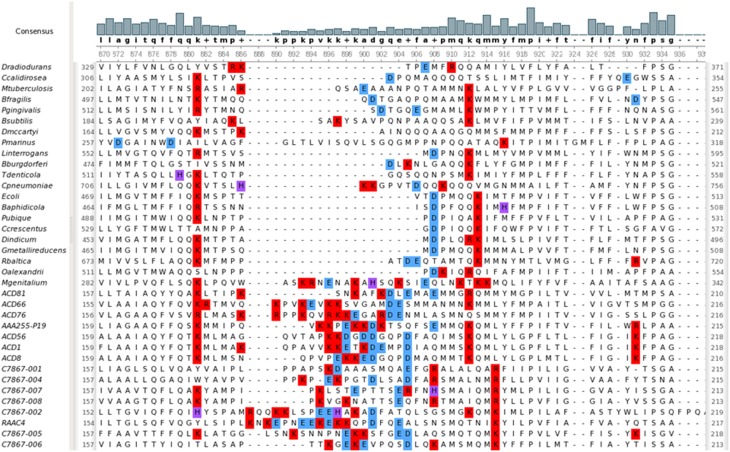
**YidC sequences from Parcubacteria have a unique region of charged residues**. Positively charged residues at neutral pH are colored red; negatively charged residues are colored blue; histidine residues, which are positively charged below pH 6.0, are colored purple. The display was generated using UGENE (Okonechnikov et al., 2012).

Signal recognition particle (SRP) binds the signal peptide sequence of nascent proteins and delivers them to Sec export systems at the membrane for proper trafficking (Akopian et al., 2013). It is a ribonucleoprotein, consisting of a single polypeptide (encoded by *ffh*) and the 4.5S RNA (encoded by *ffs*). Neither gene has been detected in any of the Parcubacteria genomes. Recently, it has been demonstrated that alleles of YidC from *Rhodopirellula baltica* and *Oceanicaulis alexandrii*, which contain an extended C-terminal region enriched in positively-charged amino acids, can partially complement a deletion of SRP in *E. coli* (Seitl et al., 2014). There are two forms of YidC; one is composed solely of a domain that interacts with SecD and the transmembrane segments of nascent integral membrane proteins, and the other has an additional N-terminal periplasmic domain of undetermined function. The YidC present in Parcubacteria is of the former type. Multiple sequence alignment comparing parcubacterial YidC sequences to those from model organisms including *R. baltica* and *O. alexandrii* shows that it has a novel internal region which is enriched for charged residues (both positive and negative) (Figure 6). It does not, however, have an extended positively charged region at its C-terminus. Possibly this YidC variant, in combination with the small volume of OD1 cells, is sufficient for proper protein trafficking. It is also of note that the OD1 genomes have a smaller percentage of genes with recognizable signal peptides (on average 3%) (Table 3), perhaps relaxing the requirement for efficient trafficking.

**Table 3:** **Genes with transmembrane helices and signal peptides**.

**Genome**	**%TM**	**%SP**
AAA011-A09	26.7	1.5
AAA255-P19	25.6	1.1
ACD1	30.7	4.0
ACD56	32.1	3.3
ACD66	25.6	4.4
ACD76	28.1	3.6
ACD8	32.2	2.5
ACD81	29.5	3.2
C7867-001	33.4	3.1
C7867-002	34.5	2.6
C7867-003	36.8	2.9
C7867-004	33.1	5.2
C7867-005	31.2	3.1
C7867-006	32.4	1.9
C7867-007	35.1	2.8
C7867-008	34.5	3.8
RAAC4	32.4	2.6
TM7x	29.5	2.0
IMG avg	22.9	6.9

The ubiquitous enzyme cofactors FMN and FAD are produced from riboflavin. Organisms either synthesize riboflavin *de novo* or import riboflavin and transform it first to FMN via riboflavin kinase (RibK) and then to FAD via FAD synthetase (RibL) (Bacher et al., 2000). In some organisms these activities are joined in one protein (RibF). The capability to both synthesize riboflavin and process it to FMN and FAD is observed in organisms with reduced genomes, including both obligate endosymbionts (Fraser et al., 1995; Shigenobu et al., 2000; Bentley et al., 2003) and free-living organisms (Giovannoni et al., 2005). All Parcubacterial genomes examined lack the genes required to synthesize riboflavin and the *ribKL/ribF* gene(s). In addition, no putative riboflavin transporters were identified in the genomes. Frequently, transporters and other genes involved in riboflavin processing are transcriptionally regulated by riboswitches (Winkler et al., 2002). No riboflavin riboswitches could be identified in any of the Parcubacteria genomes using the existing Rfam model. One possibility is that OD1 does not require FMN or FAD, and thus has lost the ability to make them. A search of the genomic complements for enzymes known to require FAD or FMN or flavin as a cofactor, however, turned up the well-conserved genes *murB* (found in 13 genomes) and *trxB* (16 genomes). Comparison of the OD1 MurB sequences to those of known structure indicates that the FAD-binding domain, which resides in the amino-terminal half of the protein, is conserved, suggesting FAD is a required cofactor for these enzymes (data not shown).

Other missing functions are more difficult to evaluate solely through analysis of the sequence data available. For example, the ribosomal small subunit methyltransferase G (*gidB*) specifically methylates position N7 of G527 on the 16S rRNA (Okamoto et al., 2007). Loop 530, in which G527 resides, is highly conserved and is important for ribosome accuracy (O'Connor et al., 1992; Van Ryk and Dahlberg, 1995). It is also a site of streptomycin binding (Melancon et al., 1988). In *Mycobacterium tuberculosis*, loss of GidB activity confers low-level resistance to streptomycin (Wong et al., 2011). Perhaps compensatory mutations in Loop 530 obviate the need for *gidB*; however this is impossible to evaluate in our dataset because 16S rRNA genes (due to their conserved sequence) are very difficult to recover from metagenomes and link to genomic bins. Ribosome silencing factor (also known as YbeB) (Hauser et al., 2012), which regulates translation by preventing association of the 50S and 30S ribosomal subunits also was not identified in the Parcubacteria. This activity is absent in symbionts and pathogens with reduced genomes such as *Buchnera, Mycoplasma, Tropheryma*, and *Wigglesworthia*, perhaps suggesting commonalities in their lifestyles in which this type of regulation is not advantageous. The extreme genome reduction within the Parcubacteria could explain the poor representation of other CSCGs. Although the absence of *guaB* is not unexpected considering the general lack of *de novo* nucleotide synthesis genes, many nucleotide interconversion genes are present, so it is puzzling that cytidylate kinase (*cmk*), guanylate kinase (*gmk*), and thymidylate kinase (*tmk*) are poorly represented while adenylate kinase (*adk*) is well-represented. Adenylate kinase has been shown to be flexible in function, partially compensating for loss of nucleoside diphosphate kinase in *E. coli* (Lu and Inouye, 1996). Perhaps relaxation of specificity within the structure of the protein allows a broad nucleoside kinase activity.

### The OD1 pan-genome

**Figure 7:**
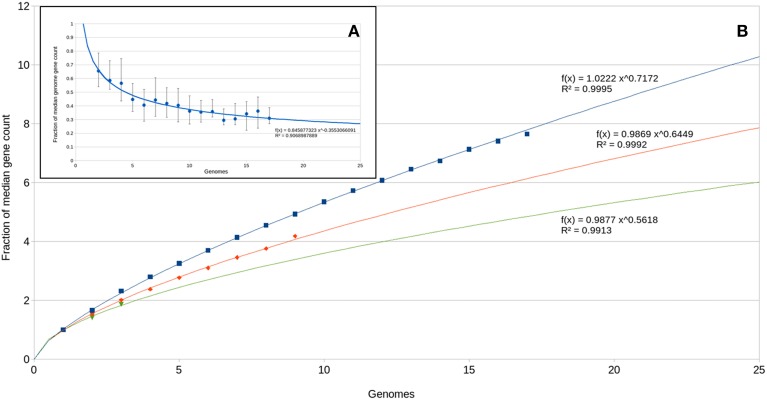
**Pan-omics analysis**. **(A)** New genes identified as a function of number of genomes analyzed. A power regression curve is fitted to the data. **(B)** Total pan-genome gene count as a function of genomes analyzed. Power regression curves are fitted to the data. Blue, all Parcubacterial genomes; red, C7867 subgroup genomes; green, subgroup OD1-i genomes.

The large number of unique genes per genome suggests a large and open pan genome. We assessed new gene and total gene accumulation as a function of genomes analyzed (Figure 7). The number of new genes identified per genome added approaches 20% of the median gene complement (Figure 7A). This level of novelty between genomes drives a near linear increase in pan-genome size as a function of genome count (Figure 7B). While this type of analysis has typically been performed on intraspecies datasets (Tettelin et al., 2005; Grote et al., 2012), its use is being expanded to higher phylogenetic levels to examine evolutionary signals (Zhang and Sievert, 2014). Potential evolutionary histories that would result in this gene distribution include: (1) an ancestral genome that was significantly larger with a broad diversity of genes and over time, descendent lineages lost different sets of genes depending on local selective forces; and (2) the ancestral genome was not significantly larger and descendent lineages acquired various genes that provided selective advantage under local conditions. There is not strong evidence for either of these options. With the apparent size of the pan genome approaching 10,000 genes, it seems unlikely that any ancestral genome contained all genes in the described pan-genome; on the other hand, newly acquired genes frequently (but not always) have a detectably different nucleotide usage signature than the rest of the genome. Over time that signature will decay to become consistent with the rest of the genome. Nucleotide skew analysis did not indicate newly acquired genes within the OD1 genomes (data not shown), and thus any acquisition of adaptive genes either occurred long ago (relative to the mutation rate) or the adaptive genes came from a donor with a similar nucleotide usage.

### Streamlining or symbiosis?

Extremely small genomes have been observed to be associated with two distinct lifestyles: free-living and streamlined to efficiently perform a limited range of metabolic activities within an oligotrophic environment, and commensal/symbiotic/parasitic in which the intimate association with the host allows the development of metabolic dependency through deletional mutation. Genome “streamlining,” as is seen in the *Pelagibacteriales* and *Prochlorococcus* is thought to be driven by large effective population sizes (N_e_) and selection for efficiency of resource acquisition and use in oligotrophic environments. Conversely, the small genomes of symbionts, parasites and commensals (SPC) (McCutcheon and Moran, 2012) are proposed to result from increased drift caused by small N_e_ or relaxed selection due to the protected, metabolite-rich environment afforded by the host cell. Because different selective mechanisms drive genome reduction in the two cases, each lifestyle results in a distinct genomic signature.

Streamlined free-living organisms have very few pseudogenes, a high coding gene density (>90% of sequence) due to short intergenic spacers, and a higher percentage of the genome complement in the conserved core of genes (Giovannoni et al., 2014). The OD1 genomes analyzed here only partially match this profile. While there is little evidence of pseudogenes, coding gene density averages only ~90%, and the conserved core only comprises 18–39% of the genome complement, a typical range for organisms related at the phylum level (Grote et al., 2012).

The OD1 genomes have more similarities to SPC genomes. SPC organisms lack biosynthetic pathways for essential metabolites such as amino acids, nucleotides, and vitamins; obligate endosymbionts in addition lack genes for fatty acid, peptidoglycan, and phospholipid synthesis. A reduced complement of DNA repair genes is also indicative of an SPC lifestyle, and could be a mechanism by which the rate of drift increases in these organisms (Moran et al., 2008). The OD1 genomes lack most biosynthetic pathways for amino acids, nucleotides, and cofactors and also do not have obvious salvage systems or large numbers of transporters. None of the OD1 possesses genes for fatty acid or phospholipid biosynthesis, but peptidoglycan biosynthesis is part of the core. Only ~4.5% of their gene complements is devoted to DNA repair and repair genes in the core are mostly involved in recombinational repair. The remainder of the repair gene complement varies; for example, A/G-specific adenine glycosylase and uracil-DNA glycosylase were only identified in the genomes from the oxic environments, while apurinic endonuclease (*nfo*) was only identified in the OD1-i group genomes. These genome signatures suggest that the Parcubacteria are symbionts, and acquire many fundamental metabolites from a partner organism through close contact.

SPC organisms must, of course, have cellular machinery for contacting and associating with their partner/prey/host. The presence of a type IV pilus (T4P) operon in the OD1 core genome provides a possible mechanism for attachment. T4P are known to be involved in cell-cell contact in a diverse set of systems. *Bdellovibrio* has been shown to require T4P to capture prey bacteria (Chanyi and Koval, 2014); T4P are required for *Francisella* virulence in mammals (Forslund et al., 2010); and *Acidovorax* uses a T4P for virulence in plants (Bahar et al., 2009). Curiously, one gene missing from the T4P operon is for the outer membrane secretin PilQ. This absence is typical of organisms lacking an outer membrane (Melville and Craig, 2013), however it has been reported that OD1 organisms have an outer membrane (Gong et al., 2014). The OD1 genomes also have unique large (>1500 aa) proteins containing beta-sheet forming domains and internal repeats, features indicative of adhesins.

One potential model for how OD1 could interact with a partner is TM7x, a strain of candidate division TM7 (*Saccharibacteria*), which has recently been shown to be an obligate ectosymbiont or parasite of *Actinomyces odontolyticus* XH001 (He et al., 2015). The TM7x genome shares many of the characteristics of the OD1 described above, including a lack of biosynthetic pathways for amino acids, nucleotides and cofactors and a Type IV pilus operon, and also shares a high proportion of proteins with transmembrane helices and a low proportion of genes with signal peptides (Table 3). Growth of TM7x required the presence of XH001, and microscopy revealed multiple TM7x cells attached around the periphery of the XH001 cells. TM7x negatively impacts the viability of XH001 suggesting the relationship is parasitic. An OD1 organism, *Candidatus* Sonnebornia yantaiensis, has been identified within *Paramecium bursaria* (Gong et al., 2014). Inside the eukaryotic cell, it was usually associated with the perialgal membrane surrounding *Chlorella* cells, an algal endosymbiont of paramecia. This may indicate a three-way association, or possibly *C*. S. yantaiensis is a parasite of *Chlorella* that is internalized by association.

An ectosymbiotic or parasitic lifestyle could explain some features of the OD1 genomes like the lack of many biosynthetic pathways and absence of a clear electron transport system, as is demonstrated by another bacterial epibiotic system, *Chlorochromatium aggregatum*. This consortium is composed of multiple cyanobacterial *Chlorobium chromatii* cells attached to the surface of a betaproteobacterium *Candiatus* Symbiobacter mobilis (Overmann, 2010). Similar to the OD1 genomes, *C*. S. mobilis lacks quinone biosynthesis pathways although it has proteins, such as cytochrome bd I oxidase and succinate dehydrogenase, that require quinone (Liu et al., 2013). It has been proposed that “periplasmic tubules” connecting the periplasms of the two organisms (Wanner et al., 2008) allow transfer of quinones or quinone precursors and sharing of proton motive force. Thus OD1 species could be obtaining nutrients, metabolites and energy directly from the host cells through such a mechanism. The great diversity in the sparse energy metabolism genes found in the OD1 genomes could be the manifestation of selection for complementary functions to the host organisms.

We have identified multiple OD1 taxa within an oxic groundwater environment and reconstructed partial and near-complete genomes for eight. Similar to previously reported OD1 studies, the genomes are very small and do not include genes for biosynthesis of nucleotides, amino acids, fatty acids, and other cofactors like quinones and flavins. Despite this extreme biosynthetic deficiency, there is also a dearth of transporters. Previous works have identified OD1 exclusively in anaerobic environments (Elshahed et al., 2003; Miyoshi et al., 2005; Borrel et al., 2010; Peura et al., 2012). Some components of electron transport chains were identified in only 3 of the 8 genomes reconstructed from the oxic groundwater metagenome sample, suggesting a disconnection between the oxic environment from which the genomes originated and OD1 cellular metabolism. The number and diversity of genes within the “flexible” genome (i.e., the set of genes not in the conserved core) suggests an evolutionary history in which species have parsimoniously adapted to a wide range of nutritional constraints, resulting in diverse yet reduced genomes. It is interesting to note that despite this large variation in gene content, the overall life history of the phylum appears consistent: the fermentation-based lifestyle and absence of biosynthetic pathways for cellular components persist within this large available genetic space. This suggests a stable and robust common mechanism for acquiring energy, nutrients, and metabolites. The genome features described herein compare favorably to those that are common among symbiotic, commensal, and parasitic organisms. Several candidate phyla for which genome sequence has been determined appear to have reduced genomes. The broad distribution of these phyla observed in community survey analyses could indicate that a SCP lifestyle is far more prevalent among bacteria than previously thought. This would have profound implications for nutrient cycles, energy recycling, and community dynamics.

### Conflict of interest statement

The authors declare that the research was conducted in the absence of any commercial or financial relationships that could be construed as a potential conflict of interest.

## References

[B1] Akopian D. Shen K. Zhang X. Shan S. O. (2013). Signal recognition particle: an essential protein-targeting machine. Annu. Rev. Biochem. 82, 693–721. 10.1146/annurev-biochem-072711-164732 23414305 PMC3805129

[B2] Alexeyenko A. Tamas I. Liu G. Sonnhammer E. (2006). Automatic clustering of orthologs and inparalogs shared by multiple proteomes. Bioinformatics 22, E9–E15. 10.1093/bioinformatics/btl213 16873526

[B3] Aziz R. K. Bartels D. Best A. A. DeJongh M. Disz T. Edwards R. A.. (2008). The RAST server: rapid annotations using subsystems technology. BMC Genomics 9:75. 10.1186/1471-2164-9-75 18261238 PMC2265698

[B4] Bacher A. Eberhardt S. Fischer M. Kis K. Richter G. (2000). Biosynthesis of vitamin b2 (riboflavin). Annu. Rev. Nutr. 20, 153–167. 10.1146/annurev.nutr.20.1.153 10940330

[B5] Bahar O. Goffer T. Burdman S. (2009). Type IV Pili are required for virulence, twitching motility, and biofilm formation of acidovorax avenae subsp. Citrulli. Mol. Plant Microbe Interact. 22, 909–920. 10.1094/MPMI-22-8-0909 19589067

[B6] Bentley S. D. Maiwald M. Murphy L. D. Pallen M. J. Yeats C. A. Dover L. G.. (2003). Sequencing and analysis of the genome of the Whipple's disease bacterium *Tropheryma whipplei*. Lancet 361, 637–644. 10.1016/S0140-6736(03)12597-4 12606174

[B7] Bolger A. M. Lohse M. Usadel B. (2014). Trimmomatic: a flexible trimmer for Illumina sequence data. Bioinformatics 30, 2114–2120. 10.1093/bioinformatics/btu170 24695404 PMC4103590

[B8] Borisov V. B. Gennis R. B. Hemp J. Verkhovsky M. I. (2011). The cytochrome bd respiratory oxygen reductases. Biochim. Biophys. Acta 1807, 1398–1413. 10.1016/j.bbabio.2011.06.016 21756872 PMC3171616

[B9] Borrel G. Lehours A. C. Bardot C. Bailly X. Fonty G. (2010). Members of candidate divisions OP11, OD1 and SR1 are widespread along the water column of the meromictic Lake Pavin (France). Arch. Microbiol. 192, 559–567. 10.1007/s00203-010-0578-4 20495786

[B10] Bostrom K. H. Simu K. Hagstrom A. Riemann L. (2004). Optimization of DNA extraction for quantitative marine bacterioplankton community analysis. Limnol. Oceanogr. Methods 2, 365–373. 10.4319/lom.2004.2.365

[B11] Camacho C. Coulouris G. Avagyan V. Ma N. Papadopoulos J. Bealer K.. (2009). BLAST+: architecture and applications. BMC Bioinformatics 10:421. 10.1186/1471-2105-10-421 20003500 PMC2803857

[B12] Carpousis A. J. (2007). The RNA degradosome of *Escherichia coli*: an mRNA-degrading machine assembled on RNase E. Annu. Rev. Microbiol. 61, 71–87. 10.1146/annurev.micro.61.080706.093440 17447862

[B13] Chan P. P. Lowe T. M. (2009). GtRNAdb: a database of transfer RNA genes detected in genomic sequence. Nucleic Acids Res. 37, D93–D97. 10.1093/nar/gkn787 18984615 PMC2686519

[B14] Chanyi R. M. Koval S. F. (2014). Role of type IV pili in predation by *Bdellovibrio bacteriovorus*. PLoS ONE 9:e113404. 10.1371/journal.pone.0113404 25409535 PMC4237445

[B15] Daub J. Eberhardt R. Y. Tate J. G. Burge S. W. (2015). Rfam: annotating families of non-coding RNA sequences. Methods Mol. Biol. 1269, 349–363. 10.1007/978-1-4939-2291-8_22 25577390

[B16] Dick G. J. Andersson A. F. Baker B. J. Simmons S. L. Thomas B. C. Yelton A. P.. (2009). Community-wide analysis of microbial genome sequence signatures. Genome Biol. 10:R85. 10.1186/gb-2009-10-8-r85 19698104 PMC2745766

[B17] Dinamarca M. A. Ruiz-Manzano A. Rojo F. (2002). Inactivation of cytochrome o ubiquinol oxidase relieves catabolic repression of the *Pseudomonas putida* GPo1 alkane degradation pathway. J. Bacteriol. 184, 3785–3793. 10.1128/JB.184.14.3785-3793.2002 12081947 PMC135178

[B18] Eddy S. R. (2011). Accelerated profile HMM searches. PLoS Comput. Biol. 7:e1002195. 10.1371/journal.pcbi.1002195 22039361 PMC3197634

[B19] Elshahed M. S. Najar F. Z. Aycock M. Qu C. Roe B. A. Krumholz L. R. (2005). Metagenomic analysis of the microbial community at Zodletone Spring (Oklahoma): insights into the genome of a member of the novel candidate division OD1. Appl. Environ. Microbiol. 71, 7598–7602. 10.1128/AEM.71.11.7598-7602.2005 16269812 PMC1287674

[B20] Elshahed M. S. Senko J. M. Najar F. Z. Kenton S. M. Roe B. A. Dewers T. A.. (2003). Bacterial diversity and sulfur cycling in a mesophilic sulfide-rich spring. Appl. Environ. Microbiol. 69, 5609–5621. 10.1128/AEM.69.9.5609-5621.2003 12957951 PMC194924

[B21] Enright A. J. Van Dongen S. Ouzounis C. A. (2002). An efficient algorithm for large-scale detection of protein families. Nucleic Acids Res. 30, 1575–1584. 10.1093/nar/30.7.1575 11917018 PMC101833

[B22] Finn R. D. Bateman A. Clements J. Coggill P. Eberhardt R. Eddy S. R.. (2014). Pfam: the protein families database. Nucleic Acids Res. 42, D222–D230. 10.1093/nar/gkt1223 24288371 PMC3965110

[B23] Forslund A. L. Salomonsson E. N. Golovliov I. Kuoppa K. Michell S. Titball R.. (2010). The type IV pilin, PilA, is required for full virulence of *Francisella tularensis* subspecies tularensis. BMC Microbiol. 10:227. 10.1186/1471-2180-10-227 20796283 PMC2941502

[B24] Fraser C. M. Gocayne J. D. White O. Adams M. D. Clayton R. A. Fleischmann R. D.. (1995). The minimal gene complement of *Mycoplasma genitalium*. Science 270, 397–403. 10.1126/science.270.5235.397 7569993

[B25] Giovannoni S. J. Thrash J. C. Temperton B. (2014). Implications of streamlining theory for microbial ecology. ISME J. 8, 1553–1565. 10.1038/ismej.2014.60 24739623 PMC4817614

[B26] Giovannoni S. J. Tripp H. J. Givan S. Podar M. Vergin K. L. Baptista D.. (2005). Genome streamlining in a cosmopolitan oceanic bacterium. Science 309, 1242–1245. 10.1126/science.1114057 16109880

[B27] Gong J. Qing Y. Guo X. Warren A. (2014). “Candidatus Sonnebornia yantaiensis”, a member of candidate division OD1, as intracellular bacteria of the ciliated protist *Paramecium bursaria* (Ciliophora, Oligohymenophorea). Syst. Appl. Microbiol. 37, 35–41. 10.1016/j.syapm.2013.08.007 24231291

[B28] Gorna M. W. Carpousis A. J. Luisi B. F. (2012). From conformational chaos to robust regulation: the structure and function of the multi-enzyme RNA degradosome. Q. Rev. Biophys. 45, 105–145. 10.1017/S003358351100014X 22169164

[B29] Grote J. Thrash J. C. Huggett M. J. Landry Z. C. Carini P. Giovannoni S. J.. (2012). Streamlining and core genome conservation among highly divergent members of the SAR11 Clade. mBio 3, 1–13. 10.1128/mBio.00252-12 22991429 PMC3448164

[B30] Haft D. H. Selengut J. D. Richter R. A. Harkins D. Basu M. K. Beck E. (2013). TIGRFAMs and genome properties in 2013. Nucleic Acids Res. 41, D387–D395. 10.1093/nar/gks1234 23197656 PMC3531188

[B31] Harris J. K. Kelley S. T. Pace N. R. (2004). New perspective on uncultured bacterial phylogenetic division OP11. Appl. Environ. Microbiol. 70, 845–849. 10.1128/AEM.70.2.845-849.2004 14766563 PMC348892

[B32] Hauser R. Pech M. Kijek J. Yamamoto H. Titz B. Naeve F.. (2012). RsfA (YbeB) proteins are conserved ribosomal silencing factors. PLoS Genet. 8:e1002815. 10.1371/journal.pgen.1002815 22829778 PMC3400551

[B33] He X. McLean J. S. Edlund A. Yooseph S. Hall A. P. Liu S. Y.. (2015). Cultivation of a human-associated TM7 phylotype reveals a reduced genome and epibiotic parasitic lifestyle. Proc. Natl. Acad. Sci. U.S.A. 112, 244–249. 10.1073/pnas.1419038112 25535390 PMC4291631

[B34] Hyatt D. Chen G. L. LoCascio P. F. Land M. L. Larimer F. W. Hauser L. J. (2010). Prodigal: prokaryotic gene recognition and translation initiation site identification. BMC Bioinformatics 11:119. 10.1186/1471-2105-11-119 20211023 PMC2848648

[B35] Kantor R. S. Wrighton K. C. Handley K. M. Sharon I. Hug L. A. Castelle C. J.. (2013). Small genomes and sparse metabolisms of sediment-associated bacteria from four candidate phyla. MBio 4, e00708–e00713. 10.1128/mBio.00708-13 24149512 PMC3812714

[B36] Katoh K. Standley D. M. (2014). MAFFT: iterative refinement and additional methods. Methods Mol. Biol. 1079, 131–146. 10.1007/978-1-62703-646-7_8 24170399

[B37] Langmead B. Salzberg S. L. (2012). Fast gapped-read alignment with Bowtie 2. Nat. Methods 9, 357–359. 10.1038/nmeth.1923 22388286 PMC3322381

[B38] Li H. Handsaker B. Wysoker A. Fennell T. Ruan J. Homer N.. (2009). The sequence Alignment/Map format and SAMtools. Bioinformatics 25, 2078–2079. 10.1093/bioinformatics/btp352 19505943 PMC2723002

[B39] Li Z. Deutscher M. P. (1994). The role of individual exoribonucleases in processing at the 3′ end of *Escherichia coli* tRNA precursors. J. Biol. Chem. 269, 6064–6071. 7509797

[B40] Lin X. Kennedy D. Fredrickson J. Bjornstad B. Konopka A. (2012a). Vertical stratification of subsurface microbial community composition across geological formations at the Hanford Site. Environ. Microbiol. 14, 414–425. 10.1111/j.1462-2920.2011.02659.x 22122741

[B41] Lin X. McKinley J. Resch C. T. Kaluzny R. Lauber C. L. Fredrickson J.. (2012b). Spatial and temporal dynamics of the microbial community in the Hanford unconfined aquifer. ISME J. 6, 1665–1676. 10.1038/ismej.2012.26 22456444 PMC3498919

[B42] Liu K. Linder C. R. Warnow T. (2011). RAxML and FastTree: comparing two methods for large-scale maximum likelihood phylogeny estimation. PLoS ONE 6:e27731. 10.1371/journal.pone.0027731 22132132 PMC3221724

[B43] Liu Z. Muller J. Li T. Alvey R. M. Vogl K. Frigaard N. U.. (2013). Genomic analysis reveals key aspects of prokaryotic symbiosis in the phototrophic consortium “*Chlorochromatium aggregatum*”. Genome Biol. 14:R127. 10.1186/gb-2013-14-11-r127 24267588 PMC4053972

[B44] Lowe T. M. Eddy S. R. (1997). tRNAscan-SE: a program for improved detection of transfer RNA genes in genomic sequence. Nucleic Acids Res. 25, 955–964. 10.1093/nar/25.5.0955 9023104 PMC146525

[B45] Lu Q. Inouye M. (1996). Adenylate kinase complements nucleoside diphosphate kinase deficiency in nucleotide metabolism. Proc. Natl. Acad. Sci. U.S.A. 93, 5720–5725. 10.1073/pnas.93.12.5720 8650159 PMC39127

[B46] Machnicka M. A. Milanowska K. Osman Oglou O. Purta E. Kurkowska M. Olchowik A.. (2013). MODOMICS: a database of RNA modification pathways–2013 update. Nucleic Acids Res. 41, D262–D267. 10.1093/nar/gks1007 23118484 PMC3531130

[B47] McCutcheon J. P. Moran N. A. (2012). Extreme genome reduction in symbiotic bacteria. Nat. Rev. Microbiol. 10, 13–26. 10.1038/nrmicro2670 22064560

[B48] Melancon P. Lemieux C. Brakier-Gingras L. (1988). A mutation in the 530 loop of *Escherichia coli* 16S ribosomal RNA causes resistance to streptomycin. Nucleic Acids Res. 16, 9631–9639. 10.1093/nar/16.20.9631 3054810 PMC338768

[B49] Melville S. Craig L. (2013). Type IV pili in Gram-positive bacteria. Microbiol. Mol. Biol. Rev. 77, 323–341. 10.1128/MMBR.00063-12 24006467 PMC3811610

[B50] Miyoshi T. Iwatsuki T. Naganuma T. (2005). Phylogenetic characterization of 16S rRNA gene clones from deep-groundwater microorganisms that pass through 0.2-micrometer-pore-size filters. Appl. Environ. Microbiol. 71, 1084–1088. 10.1128/AEM.71.2.1084-1088.2005 15691970 PMC546738

[B51] Moran N. A. McCutcheon J. P. Nakabachi A. (2008). Genomics and evolution of heritable bacterial symbionts. Annu. Rev. Genet. 42, 165–190. 10.1146/annurev.genet.41.110306.130119 18983256

[B52] O'Connor M. Goringer H. U. Dahlberg A. E. (1992). A ribosomal ambiguity mutation in the 530 loop of *E. coli* 16S rRNA. Nucleic Acids Res. 20, 4221–4227. 10.1093/nar/20.16.4221 1380697 PMC334129

[B53] Okamoto S. Tamaru A. Nakajima C. Nishimura K. Tanaka Y. Tokuyama S.. (2007). Loss of a conserved 7-methylguanosine modification in 16S rRNA confers low-level streptomycin resistance in bacteria. Mol. Microbiol. 63, 1096–1106. 10.1111/j.1365-2958.2006.05585.x 17238915

[B54] Okonechnikov K. Golosova O. Fursov M. Team U. (2012). Unipro UGENE: a unified bioinformatics toolkit. Bioinformatics 28, 1166–1167. 10.1093/bioinformatics/bts091 22368248

[B55] Overbeek R. Olson R. Pusch G. D. Olsen G. J. Davis J. J. Disz T.. (2014). The SEED and the Rapid Annotation of microbial genomes using Subsystems Technology (RAST). Nucleic Acids Res. 42, D206–D214. 10.1093/nar/gkt1226 24293654 PMC3965101

[B56] Overmann J. (2010). The phototrophic consortium “*Chlorochromatium aggregatum*” - a model for bacterial heterologous multicellularity. Adv. Exp. Med. Biol. 675, 15–29. 10.1007/978-1-4419-1528-3_2 20532733

[B57] Peng Y. Leung H. C. Yiu S. M. Chin F. Y. (2012). IDBA-UD: a de novo assembler for single-cell and metagenomic sequencing data with highly uneven depth. Bioinformatics 28, 1420–1428. 10.1093/bioinformatics/bts174 22495754

[B58] Peura S. Eiler A. Bertilsson S. Nykanen H. Tiirola M. Jones R. I. (2012). Distinct and diverse anaerobic bacterial communities in boreal lakes dominated by candidate division OD1. ISME J. 6, 1640–1652. 10.1038/ismej.2012.21 22418623 PMC3498924

[B59] Rankin D. J. Rocha E. P. Brown S. P. (2011). What traits are carried on mobile genetic elements, and why? Heredity (Edinb.) 106, 1–10. 10.1038/hdy.2010.24 20332804 PMC3183850

[B60] Rinke C. Schwientek P. Sczyrba A. Ivanova N. N. Anderson I. J. Cheng J. F.. (2013). Insights into the phylogeny and coding potential of microbial dark matter. Nature 499, 431–437. 10.1038/nature12352 23851394

[B61] Seitl I. Wickles S. Beckmann R. Kuhn A. Kiefer D. (2014). The C-terminal regions of YidC from *Rhodopirellula baltica* and *Oceanicaulis alexandrii* bind to ribosomes and partially substitute for SRP receptor function in *Escherichia coli*. Mol. Microbiol. 91, 408–421. 10.1111/mmi.12465 24261830

[B62] Shigenobu S. Watanabe H. Hattori M. Sakaki Y. Ishikawa H. (2000). Genome sequence of the endocellular bacterial symbiont of aphids Buchnera sp. APS. Nature 407, 81–86. 10.1038/35024074 10993077

[B63] Tettelin H. Masignani V. Cieslewicz M. J. Donati C. Medini D. Ward N. L.. (2005). Genome analysis of multiple pathogenic isolates of *Streptococcus agalactiae*: implications for the microbial “pan-genome.” Proc. Natl. Acad. Sci. U.S.A. 102, 13950–13955. 10.1073/pnas.0506758102 16172379 PMC1216834

[B64] Thorvaldsdottir H. Robinson J. T. Mesirov J. P. (2013). Integrative Genomics Viewer (IGV): high-performance genomics data visualization and exploration. Brief. Bioinformatics 14, 178–192. 10.1093/bib/bbs017 22517427 PMC3603213

[B65] VanOrsdel C. E. Bhatt S. Allen R. J. Brenner E. P. Hobson J. J. Jamil A.. (2013). The *Escherichia coli* CydX protein is a member of the CydAB cytochrome bd oxidase complex and is required for cytochrome bd oxidase activity. J. Bacteriol. 195, 3640–3650. 10.1128/JB.00324-13 23749980 PMC3754557

[B66] Van Ryk D. I. Dahlberg A. E. (1995). Structural changes in the 530 loop of *Escherichia coli* 16S rRNA in mutants with impaired translational fidelity. Nucleic Acids Res. 23, 3563–3570. 10.1093/nar/23.17.3563 7567470 PMC307238

[B67] Wanner G. Vogl K. Overmann J. (2008). Ultrastructural characterization of the prokaryotic symbiosis in “*Chlorochromatium aggregatum*”. J. Bacteriol. 190, 3721–3730. 10.1128/JB.00027-08 18344357 PMC2394997

[B68] Winkler W. C. Cohen-Chalamish S. Breaker R. R. (2002). An mRNA structure that controls gene expression by binding FMN. Proc. Natl. Acad. Sci. U.S.A. 99, 15908–15913. 10.1073/pnas.212628899 12456892 PMC138538

[B69] Wong S. Y. Lee J. S. Kwak H. K. Via L. E. Boshoff H. I. Barry C. E. III. (2011). Mutations in gidB confer low-level streptomycin resistance in *Mycobacterium tuberculosis*. Antimicrob. Agents Chemother. 55, 2515–2522. 10.1128/AAC.01814-10 21444711 PMC3101441

[B70] Wrighton K. C. Castelle C. J. Wilkins M. J. Hug L. A. Sharon I. Thomas B. C.. (2014). Metabolic interdependencies between phylogenetically novel fermenters and respiratory organisms in an unconfined aquifer. ISME J. 8, 1452–1463. 10.1038/ismej.2013.249 24621521 PMC4069391

[B71] Wrighton K. C. Thomas B. C. Sharon I. Miller C. S. Castelle C. J. VerBerkmoes N. C.. (2012). Fermentation, hydrogen, and sulfur metabolism in multiple uncultivated bacterial phyla. Science 337, 1661–1665. 10.1126/science.1224041 23019650

[B72] Wu M. Scott A. J. (2012). Phylogenomic analysis of bacterial and archaeal sequences with AMPHORA2. Bioinformatics 28, 1033–1034. 10.1093/bioinformatics/bts079 22332237

[B73] Zachara J. M. Long P. E. Bargar J. Davis J. A. Fox P. Fredrickson J. K.. (2013). Persistence of uranium groundwater plumes: contrasting mechanisms at two DOE sites in the groundwater-river interaction zone. J. Contam. Hydrol. 147, 45–72. 10.1016/j.jconhyd.2013.02.001 23500840

[B74] Zhang Y. Sievert S. M. (2014). Pan-genome analyses identify lineage- and niche-specific markers of evolution and adaptation in Epsilonproteobacteria. Front. Microbiol. 5:110. 10.3389/fmicb.2014.00110 24678308 PMC3958643

