# Supplementary figures and images for: The reduced genomes of Parcubacteria (OD1) contain signatures of a symbiotic lifestyle
Source: Front Microbiol. 2015 Jul 21;6:713. doi: 10.3389/fmicb.2015.00713 (PMC4508563; doi:10.3389/fmicb.2015.00713)

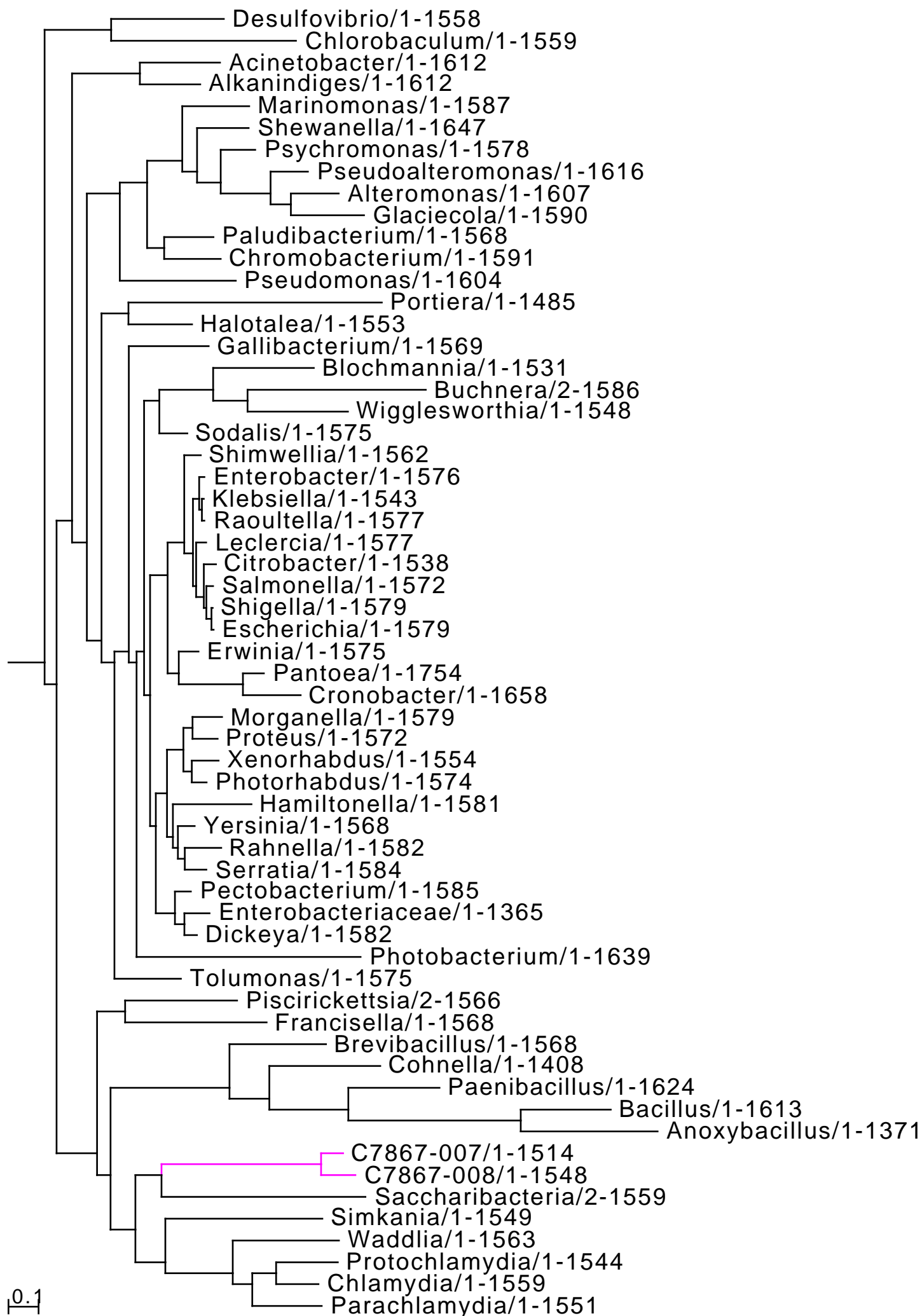

Supplement: Supplementary file 3 [file Image1.PDF]

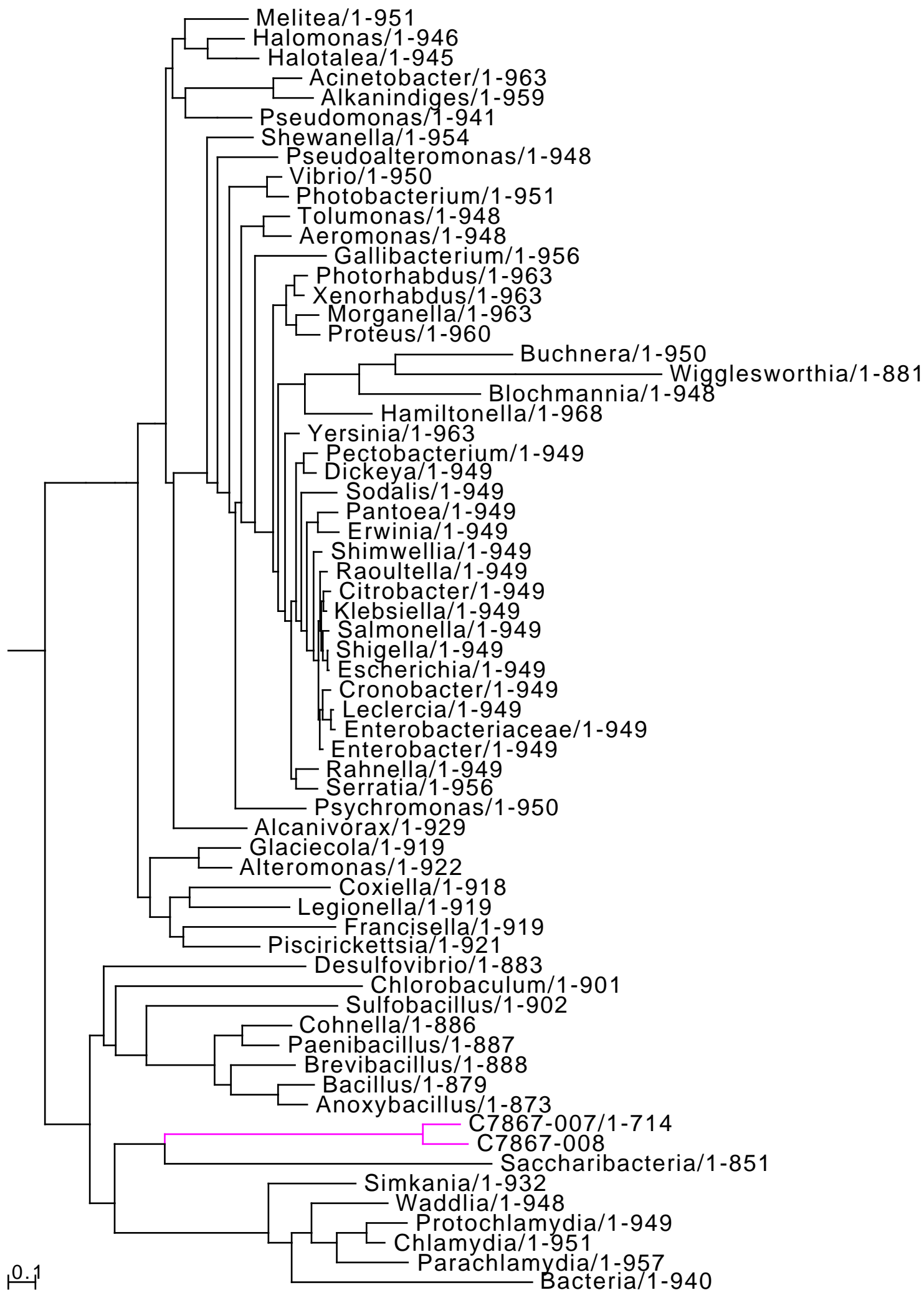

Supplement: Supplementary file 4 [file Image2.PDF]
